# Genome mining and OSMAC strategies unveil diverse secondary metabolites from the endophytic fungus *Diaporthe kyushuensis* ZMU-48-1 with antifungal activity

**DOI:** 10.3389/fmicb.2025.1604639

**Published:** 2025-06-10

**Authors:** Jiaqi Zheng, Haiwen Wang, Xijing Wang, Siyu Zeng, Siwen Yuan, Tianpeng Yin

**Affiliations:** School of Bioengineering, Zunyi Medical University, Zhuhai, Guangdong, China

**Keywords:** *Diaporthe kyushuensis*, genome mining, natural products, biosynthetic gene clusters, antifungal activity

## Abstract

Fungal-derived bioactive natural products are a crucial resource for drug discovery; however, under standard laboratory cultivation conditions, fungi predominantly yield known and repetitively isolated metabolites. This metabolic constraint presents a major obstacle to the discovery of structurally novel and bioactive secondary metabolites. Recent advances in whole-genome sequencing have revealed that a significant portion of fungal biosynthetic gene clusters (BGCs) remain silent or unexpressed under conventional culture conditions, underscoring the importance of activating these cryptic BGCs. In this study, we systematically explored the biosynthetic potential of the terrestrial-derived fungus *Diaporthe kyushuensis* ZMU-48-1, which was isolated from decayed leaves of *Acacia confusa* Merr., by integrating genome mining with the one-strain-many-compounds (OSMAC) strategy. Whole-genome sequencing and antiSMASH analysis identified 98 BGCs, of which approximately 60% exhibited no significant homology to known clusters, highlighting their potential novelty. The optimization of culture conditions *via* the OSMAC approach revealed that Potato Dextrose Broth (PDB) supplemented with 3% NaBr, PDB supplemented with 3% sea salt, and rice solid medium were optimal for increasing metabolite diversity. Large-scale fermentation and chromatographic separation yielded 18 structurally diverse compounds, including two novel pyrrole derivatives, kyushuenines A (**1**) and B (**2**), alongside 16 known secondary metabolites. Antifungal assays demonstrated that compound **8** exhibited activity against *Bipolaris sorokiniana* (MIC = 200 μg/mL), whereas compound **18** displayed potent inhibition of *Botryosphaeria dothidea* (MIC = 50 μg/mL), underscoring their potential as antifungal agents. These findings underscore the untapped chemical diversity of *D. kyushuensis* and its potential as a resource for drug discovery.

## 1 Introduction

Fungal-derived natural products are a crucial source for drug discovery, with the majority of clinically used drugs being derived directly or indirectly from natural products (Jakubczyk and Dussart, 2020; Newman and Cragg, 2020). These compounds exhibit diverse bioactivities, making them valuable for the development of antibiotics, anticancer agents, and other therapeutic drugs (Zhang et al., 2006; Nisa et al., 2015; Nazir et al., 2024). However, under standard laboratory cultivation conditions, fungi often produce a limited and repetitive set of metabolites, leading to the frequent rediscovery of known compounds (Rutledge and Challis, 2015; Chen J. et al., 2016; Zhang D. et al., 2023; Wu et al., 2025). This metabolic constraint presents a major obstacle to the discovery of structurally novel and bioactive secondary metabolites, thereby hindering the advancement of drug development and the exploration of new therapeutic leads.

Genome mining has revolutionized natural product discovery, offering a powerful approach to uncovering cryptic natural products in microorganisms (Harvey et al., 2015; Yee et al., 2023; Wei et al., 2025). By leveraging whole-genome sequencing, homology-based functional predictions, and advanced bioinformatics algorithms, biosynthetic gene clusters (BGCs) responsible for the production of diverse secondary metabolites can be systematically identified (Khaldi et al., 2010; Blin et al., 2019; Navarro-Muñoz et al., 2020; Kautsar et al., 2021). With over one million BGCs predicted from existing genomic databases, this approach unveils a vast reservoir of untapped natural products. Thus, genome mining serves as a pivotal tool for the discovery of novel bioactive compounds, significantly expanding the chemical space for drug development and biomedical research (Ziemert et al., 2016; Gilchrist et al., 2018; Biermann and Helfrich, 2021).

*Diaporthe* is a significant fungal genus within the family Diaporthaceae, order Diaporthales, and class Sordariomycetes (Guarnaccia et al., 2018). Predominantly isolated from diverse hosts, species of this genus are known plant pathogens capable of infecting a wide range of plant hosts (Dissanayake et al., 2020). *Diaporthe* species are predominantly identified as plant pathogens and are known for producing a variety of bioactive metabolites, including antifungal and phytotoxic compounds, which may play roles in ecological interactions and host colonization. Our group recently reported that species within the genus *Diaporthe* harbor numerous biosynthetic gene clusters (BGCs), suggesting their potential to produce a diverse array of secondary metabolites (SMs). However, to the best of our knowledge, reported SMs from *Diaporthe* have predominantly been polyketides, accounting for approximately 64% of known metabolites (Xu et al., 2021; Matio Kemkuignou et al., 2022; Yuan et al., 2022b; Matio Kemkuignou et al., 2023; Ma et al., 2024). This finding indicates that many BGCs within *Diaporthe* remain silent under typical laboratory culture conditions. Consequently, activating these cryptic BGCs represents a crucial strategy for expanding the chemical diversity of *Diaporthe*-derived SMs.

Various approaches have been demonstrated to effectively awaken silent BGCs, including one-strain-many-compounds (OSMAC) (Scherlach and Hertweck, 2006; Scherlach et al., 2010), epigenetic approaches (Fan et al., 2017; Zheng et al., 2017), heterologous expression (Biggins et al., 2011; Yuan et al., 2022a), and metabolic shunting activation (Wei et al., 2021; Ning et al., 2024; Wang and Mao, 2025). These methodologies have been shown to induce the biosynthesis of novel secondary metabolites, underscoring their potential for natural product discovery. Compared with other BGC activation strategies, the OSMAC approach is a rapid and efficient strategy for BGC activation, offering simplicity, cost-effectiveness, and the ability to induce diverse secondary metabolites without genetic manipulation. Widely employed in natural product research, it enhances chemical diversity and facilitates the discovery of novel bioactive compounds (Romano et al., 2018). By leveraging microbial metabolic plasticity, OSMAC modulates cultivation parameters such as medium composition and culture conditions (Hewage et al., 2014; Siridechakorn et al., 2017; Zhang S. et al., 2023). These perturbations trigger transcriptional reprogramming, activating cryptic BGCs and unveiling previously undetected metabolites (Liu T. et al., 2022).

In this study, the genome of *D. kyushuensis* ZMU-48-1 was sequenced and analyzed using antiSMASH, which revealed 98 BGCs, suggesting its potential to produce diverse secondary metabolites. To explore this potential, we combined genome mining with the OSMAC strategy, aiming to identify novel natural products and assess their bioactivities, particularly antifungal properties against phytopathogens.

## 2 Materials and methods

### 2.1 General experimental procedures

NMR spectra were obtained with a Bruker AVANCE III 600 MHz NMR spectrometer (Bruker, Germany) equipped with a 5 mm cryoprobe (CPP BBO600S3 BB-H&F-D-05 Z XT). Deuterated solvents, including CDCl_3_ (99.8%, atom %D), CD_3_OH (99.8%, atom %D), and DMSO-*d*_6_ (99.8%, atom %D), were purchased from Cambridge Isotope Laboratories (USA). HR-ESI-MS data were measured on an Orbitrap Fusion Lumos mass spectrometer (Thermo, United States). IR spectroscopic data were acquired on an Agilent Cary630 Fourier transform infrared spectrometer (Agilent, United States). Optical rotations were measured on an MCP 200 polarimeter (Anton Paar, Austria). Preparative HPLC was performed *via* a Waters 1,525 binary gradient pump and a Waters 2,998 photodiode array detector with an Xtimate C18 column (10 × 250 mm, 5 μm; Welch Materials), an Ultimate XB-Phenyl column (10 × 250 mm, 5 μm; Welch Materials, United States), and an ACE C_18_ column (4.6 mm × 250 mm, 5 μm; Advanced Chromatography Technology). Chromatographic grade acetonitrile and methanol were obtained from Tianjin Concord Technology (China). All preparative HPLC separations were performed with multi-wavelength UV detection at 220, 254, 275, and 310 nm. The silica gel (300–400 mesh) used for column chromatography (CC) and the GF254 plates used for thin layer chromatography (TLC) were purchased from Qingdao Haiyang Chemical Factory (China). The spots on the TLC plates were visualized under UV light or by spraying the plates with vanillin-sulfuric acid reagent followed by heating. The analytical grade chemicals used were methanol, dichloromethane, petroleum ether, ethyl acetate, and ethanol, all of which were procured from Tianjin Damao Chemical Reagent Co., Ltd. (Tianjin, China).

### 2.2 Fungal material and phylogenetic tree construction

The fungus *Diaporthe kyushuensis* ZMU-48-1 was isolated from decayed leaves of *Acacia confusa Merr.* collected in Huanglübei Mountain, Jinwan District, Zhuhai, China, in October 2023. The fungus is preserved in the Microbial Resource Bank of Zhuhai Campus, Zunyi Medical University, China. The whole-genome sequencing was conducted by Sangon Biotech Co., Ltd. (Shanghai, China). The isolation and purification of the fungal strain followed standard protocols (Kjer et al., 2010). Molecular identification was performed through DNA amplification and sequencing of the internal transcribed spacer (ITS) region.

The ITS sequence was analyzed using the BLAST algorithm against the ITS subdatabase of the NCBI nucleotide database, with the identity threshold set to >95%. The top 30 ITS sequences with the highest identity were selected for further analysis. Multiple sequence alignment was performed *via* MAFFT software, followed by manual trimming to remove ambiguous regions. A phylogenetic tree was subsequently constructed *via* FastTree software.

### 2.3 Genome sequencing and mining

The fungus *D. kyushuensis* ZMU-48-1 was cultured in 50 mL PDB medium at 28°C and 180 rpm for 6 days. Dried mycelium (20 mg) was ground in liquid nitrogen, mixed with 400 μL of Buffer Digestion and 4 μL of *β*-mercaptoethanol, and incubated at 65°C for 1 h. After adding 20 μL of RNase A, the mixture was incubated at room temperature for 5 min. Following the addition of 200 μL of Buffer PF and incubation at −20°C for 5 min, the supernatant was collected by centrifugation (10,000 rpm, 5 min). If cloudy, an equal volume of chloroform was added, and the mixture was centrifuged at 12,000 rpm. DNA was precipitated with isopropanol, washed with 75% ethanol, air-dried, and dissolved in 50 μL of TE buffer. DNA quality was assessed by 1% agarose gel electrophoresis and quantified using a Qubit fluorometer (Life Technologies).

Library preparation was performed with the Hieff NGS^®^ MaxUp II DNA Library Prep Kit for Illumina^®^. DNA (500 ng) was diluted to 130 μL, fragmented to ~500 bp using a Covaris S220, and processed for end-repair and A-tailing at 30°C for 20 min and 72°C for 20 min. Adapter ligation was performed via incubation with ligation reagents at 20°C for 15 min. Ligated DNA was purified using Hieff NGS™ DNA Selection Beads with dual-size selection. PCR amplification was carried out using 2 × Super Canace^®^ High-Fidelity Mix under the following conditions: 98°C for 1 min; 12 cycles of 98°C for 10 s, 60°C for 30 s, and 72°C for 30 s; and a final extension at 72°C for 5 min. The amplified library was purified using 0.8 × magnetic beads, washed with 80% ethanol, eluted in 30 μL of TE buffer, and stored at −20°C. Library size was confirmed by 2% agarose gel electrophoresis, and the concentration was measured using a Thermo Qubit 4.0 fluorometer for compatibility with the Illumina™ sequencing platform.

The genome of *D. kyushuensis* ZMU-48-1 was sequenced using the Illumina next-generation sequencing platform (Bolger et al., 2014). The raw sequencing data underwent quality control using Fastp to remove adapter sequences, low-quality bases (Q < 20), and reads shorter than 35 bp, yielding high-quality clean reads. Contamination assessment was performed by aligning 10,000 randomly sampled reads against the NCBI NT database. Genome assembly was conducted using SPAdes for *de novo* assembly, with GapFiller used to close assembly gaps and PrInSeS-G applied for sequence correction to address base errors and small insertion/deletion events (Bankevich et al., 2012; Boetzer and Pirovano, 2012; Koren et al., 2017). Genome characteristics, including size, heterozygosity, and repeat content, were estimated through k-mer analysis. Assembly quality and completeness were evaluated using BUSCO against the OrthoDB database (Tegenfeldt et al., 2025).

Gene prediction was performed using GlimmerHMM for coding sequences (CDS), Aragorn for tRNA identification, and RNAmmer for rRNA detection. Repeat sequences were identified and classified using RepeatMasker (Delcher et al., 1999; Majoros et al., 2004). Functional annotation was achieved by comparing predicted protein sequences against multiple databases using NCBI BLAST+ and HMMER3, including NR (non-redundant proteins), Swiss-Prot, COG (Clusters of Orthologous Groups), KEGG (Kyoto Encyclopedia of Genes and Genomes), and CAZy (Carbohydrate-Active enZYmes) (Altschul et al., 1997; Moriya et al., 2007; Massouras et al., 2010; Seemann, 2014). Virulence factors and antibiotic resistance genes were annotated using the VFDB and CARD databases, respectively (Chen L. et al., 2016). Comparative genomic analysis was conducted by extracting ITS sequences, aligning them with the NCBI NT database, and selecting sequences with ≥95% similarity. Multiple sequence alignments were performed using MAFFT, and phylogenetic trees were constructed using FastTree or RAxML (Katoh et al., 2019). Orthofinder2 was employed for pan-genome and homologous gene cluster analyses, while gene family expansion and contraction were assessed using CAFE5 (Emms and Kelly, 2019). Collinearity analysis was conducted using MUMmer and JCVI. These comprehensive analyses provide insights into the genetic characteristics and evolutionary relationships of *D. kyushuensis* ZMU-48-1.

The genome and GFF annotation files of *D. kyushuensis* ZMU-48-1 were uploaded to antiSMASH version 7.0 for a comprehensive analysis of the BGCs responsible for secondary metabolite production, enabling the identification, classification, and comparative analysis of the BGCs to gain detailed insights into the types of secondary metabolites the genome can produce (Blin et al., 2019).

### 2.4 One strain many compounds strategy

To identify the optimal medium, small-scale fermentation of *D. kyushuensis* ZMU-48-1 was performed using eleven different liquid media and one rice solid medium. The fungus was initially cultured in 200 mL PDB for 2 days at 28°C to establish a seed culture. Subsequently, the seed culture was transferred into 200 mL of each of the eleven liquid media and incubated for 14 days at 28°C with continuous shaking at 180 rpm. In parallel, fermentation was also conducted on a rice medium composed of 50 g of rice and 3% sea salt. The formulations for the eleven liquid media and one solid medium are as follows: A medium: Dissolve 1 g soluble starch, 0.5 g yeast extract, 0.2 g pancreatic peptone, 0.2 g CaCO₃, 0.004 g Fe₂(SO₄)₃·4H₂O, 0.01 g KBr, and 3 g sea salt in deionized water to a final volume of 100 mL; P medium: Dissolve 0.2 g yeast extract, 0.4 g mannitol, 0.2 g peptone, and 3 g sea salt in deionized water to a final volume of 100 mL; COB medium: Dissolve 0.5 g multivalent peptone, 0.5 g yeast extract, 0.1 g MgSO₄·7H₂O, 0.05 g KH₂PO₄, 3 g sucrose, and 3 g sea salt in deionized water to a final volume of 100 mL, and adjust the pH to 5.5; and MMD medium: Dissolve 0.5 g peptone, 0.3 g fish peptone, 2 g sucrose, 0.1 g MgSO₄, 0.2 g KH₂PO₄, and 3 g sea salt in deionized water to a final volume of 100 mL; ME medium: Dissolve 0.2 g malt extract, 2 g sucrose, 0.1 g peptone, and 3 g sea salt in deionized water to a final volume of 100 mL; Gao’s medium: Dissolve 2 g soluble starch, 0.1 g KNO₃, 0.05 g K₂HPO₄, 0.05 g MgSO₄·7H₂O, 0.001 g FeSO₄·7H₂O, and 3 g sea salt in deionized water to a final volume of 100 mL; PDB medium: Dissolve 30 g potatoes, 2 g glucose in deionized water to a final volume of 100 mL; Modified PDB medium: PDB with 3% NaBr, 3% KI, 3% sea salt, and 0.3% NaBr, respectively; Rice medium: Consist of 50 g rice and 3% sea salt. All media were sterilized at 121°C for 30 min.

For large-scale fermentation, seed cultures of *D. kyushuensis* ZMU-48-1 were prepared as described previously. Subsequently, 10 mL of the seed cultures were inoculated into 500 mL Erlenmeyer flasks containing 200 mL of PDB supplemented with 3% NaBr or PDB supplemented with 3% sea salt. The cultures were incubated for 14 days at 28°C with shaking at 180 rpm, with a total fermentation volume of 4 L for each medium. Additionally, rice medium, consisting of 2 kg of rice, was also employed for large-scale fermentation.

### 2.5 HPLC analysis of different secondary metabolites

The crude secondary metabolites were analyzed by HPLC using an ACE C_18_ column (4.6 mm × 250 mm, 5 μm) with an acetonitrile-water mobile phase at a flow rate of 3 mL/min. The gradient program was as follows: 10% acetonitrile at 0 min, increased to 50% at 15 min, increased to 100% at 30 min, maintained until 37 min, and returned to 10% at 40 min.

### 2.6 Isolation and identification of compounds

The liquid cultures were extracted with EA three times, and the mycelia were sonicated in CH_3_OH three times. These two extraction solutions were combined and evaporated under reduced pressure to obtain the corresponding crude extracts.

The rice medium was soaked in 50 L of EtOH for 7 days (three times). The extraction solutions were concentrated and then extracted with 45 L of EA to afford the crude extracts (Supplementary Figure S1).

The crude extracts of PDB medium supplemented with 3% NaCl fermentation broth (6.5 g) were separated into seven fractions (Fr. A1–Fr. A7) with a gradient silica gel CC (PE–EA, 100% to 0). Fr. A3 was further separated via preparative HPLC using an acetonitrile-water system (12% acetonitrile) on an Ultimate XB-Phenyl column (10 × 250 mm, 5 μm; Welch Materials) to obtain compounds **1** (4 mg, t_R_ = 7.2 min), **2** (7 mg, t_R_ = 7.2 min), **3** (5 mg, t_R_ = 8.6 min) and **4** (12 mg, t_R_ = 8.6 min).

The crude extracts of PDB medium supplemented with 3% NaBr fermentation broth (7.3 g) were subjected to silica gel CC with a gradient system (PE-EA, 100% to 0) to afford seven fractions (Fr. C1-Fr. C7). Fr. C3 was further separated by preparative HPLC using an acetonitrile-water system on an ACE C18 column (4.6 mm × 250 mm, 5 μm; Advanced Chromatography Technologies). Compound **13** (6 mg, t_R_ = 10.1 min) was obtained using a mobile phase of 30% acetonitrile, whereas compound **14** (5 mg, t_R_ = 7.6 min) was obtained using a mobile phase of 70% acetonitrile.

The crude extracts of fermented rice medium (66.7 g) were subjected to silica gel CC (PE-EA, 100% to 0) to afford seven fractions (Fr. B1–Fr. B7). Fr. B3 was further divided into seven fractions (Fr. B3-1–Fr. B3-7) via silica gel CC eluted with a CH_2_Cl_2_-CH_3_OH gradient system (20:1 to 2:1). Fr. B3-3 (1.2 g) was purified by preparative HPLC using an acetonitrile-water system (25% acetonitrile) on an Ultimate XB-Phenyl column to obtain compounds **5** (12 mg, t_R_ = 11.6. min), **6** (16 mg, t_R_ = 14.3 min), **7** (8 mg, t_R_ = 16.1 min), and **8** (18 mg, t_R_ = 9.2 min). Fr. B3-4 (1.6 g) was isolated via preparative HPLC using an acetonitrile-water system (17% acetonitrile) on an Ultimate XB-Phenyl column to obtain compounds **17** (11 mg, t_R_ = 8.4 min) and **18** (9 mg, t_R_ = 10.2 min). Fr. B3-5 (1.4 g) was purified via preparative HPLC using an acetonitrile-water system (17% acetonitrile) on an Xtimate C18 column (10 × 250 mm, 5 μm; Welch Materials) to afford compounds **9** (11 mg, t_R_ = 7.9 min), **10** (9 mg, t_R_ = 8.5 min), **15** (11 mg, t_R_ = 12.7 min) and **16** (7 mg, t_R_ = 14.1 min). Further purification of Fr. B6 (1.2 g, tR = 7.6 min) *via* preparative HPLC using a methanol–water system (25% methanol) on an Xtimate C18 column afforded compounds **11** (31 mg, t_R_ = 14.8 min) and **12** (26 mg, t_R_ = 16.6 min).

Kyushuenine A (**1**): white amorphous powder; IR (KBr, cm^−1^): 2926, 2,854, 2,358, 2071, 1,633, 1,396, 1,082, 599. The ^1^H and ^13^C NMR spectral data are shown in Table 1. HR-ESI-MS *m/z* 140.0679 [M + H]^+^ (calcd for C_7_H_10_NO_2_, 140.0712).

**Table 1:** ^1^H (600 MHz) and ^13^C NMR (150 MHz) data of compounds **1** and **2** in CD_3_OD.

Nos.	**1**		**2**	
	*δ*_H_ (*J* in Hz)	*δ*_C_	*δ*_H_ (Mult., *J* in Hz)	*δ*_C_
2	/	143.3 s	/	141.7 s
3	/	120.9 s	/	120.4 s
4	/	125.9 s	/	122.6 s
5	6.63 s	117.4 d	6.65 s	118.9 s
6	2.49 s	11.3 q	2.48 s	11.7 q
7	9.79 s	187.9 s	9.84 s	187.7 s
8	4.61 s	58.1 t	4.55 s	67.8 t
OCH_3_-8	/		3.36 s	57.9 q

Kyushuenine B (**2**): white amorphous powder; IR (KBr, cm^−1^): 2927, 2,857, 2,362, 2073, 1,647, 1,118, 972. The ^1^H and ^13^C NMR spectral data are shown in Table 1. HR-ESI-MS *m/z* 154.0864 [M + H]^+^ (calcd for C_8_H_12_NO_2_, 154.0868).

### 2.7 Antifungal activity

All the compounds were evaluated for their antifungal activity against *Fusarium graminearum*, *Botryosphaeria dothidea*, *Fusarium oxysporum* Schltdl*, Colletotrichum gloeosporioides*, *Valsa mali* Miyabe & G. Yamada, *Colletotrichum musae*, *Alternaria alternata*, *Colletotrichum agenarium*, and *Bipolaris sorokiniana*. The minimum inhibitory concentration (MIC) was determined using a serial dilution method (Feng and Ma, 2010). Ten microtubes were prepared, each containing 0.5 mL of liquid culture medium. A 0.5 mL aliquot of the sample solution, prepared at an initial concentration of 512 μg/mL (0.5–5% *v*/*v*, DMSO/water), was added to the first tube and thoroughly mixed. Subsequently, 0.5 mL of the solution was transferred sequentially from the first tube to the second, and this serial dilution process was continued through the ninth tube. Following the final transfer, 0.5 mL of the solution was discarded from the ninth tube to maintain a uniform volume. The tenth tube, containing only the culture medium, served as a blank control. Following dilution, 0.5 mL of a spore suspension was introduced into each tube, ensuring uniform mixing. The tubes were then incubated at 28°C for 24 h under constant conditions. Fungal growth was assessed visually, and the MIC was defined as the lowest concentration at which no observable fungal growth occurred. Carbendazim was employed as a positive control to validate the assay.

## 3 Results and discussion

### 3.1 Identification of *Diaporthe kyushuensis* ZMU-48-1

**Figure 1:**
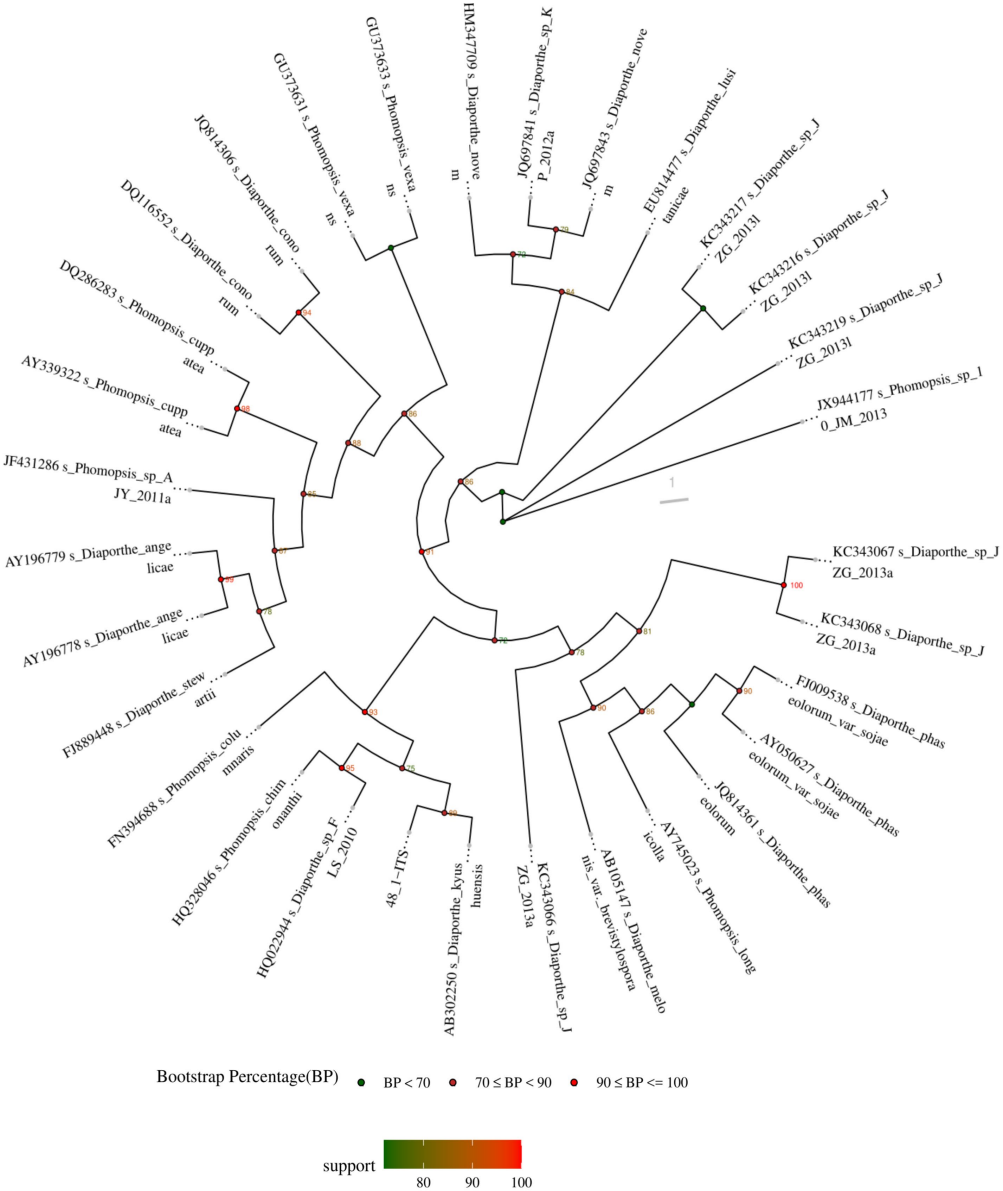
The phylogenetic tree of *D. kyushuensis* ZMU-48-1 and its closest relatives within the genus *Diaporthe* was constructed based on ITS gene sequences.

The ITS sequence of *D. kyushuensis* ZMU-48-1 was analyzed using BLAST against the NCBI database. The closest related strains were identified as *Diaporthe melonis* var. *brevistylospora* (AB105147), *Diaporthe phaseolorum* var. *sojae* (AY050627), *Diaporthe novem* (HM347709), and *Diaporthe kyushuensis* (AB302250), with sequence identities of 96.26, 96.41, 97.33, and 97.69%, respectively. Based on the BLAST results and phylogenetic analysis (Figure 1), the fungus was identified as *D. kyushuensis* ZMU-48-1. The ITS sequence of *D. kyushuensis* ZMU-48-1 has been deposited in the GenBank database with the accession number PV259067.

### 3.2 Genome and BGC analysis

Whole-genome sequencing of *D. kyushuensis* ZMU-48-1 revealed a genome size of 58.2 Mb with a GC content of 51% and low heterozygosity (0.0547%). Repetitive sequences accounted for 4.26% of the genome, primarily consisting of simple sequence repeats (0.82%) and long terminal repeat (LTR) retrotransposons from the Copia family (0.90%). Assembly using SPAdes and GapFiller produced 283 contigs, with an N50 of 594,019 bp. A total of 16,984 protein-coding genes were predicted, representing 49.04% of the genome, with an average gene length of 1,680 bp and the longest gene measuring 25,604 bp. The whole-genome sequencing data of *D. kyushuensis* ZMU-48-1 are available in NCBI’s GenBank under BioProject PRJNA1261691, GenBank accession number JBNTRE010000000, and submission ID SUB15313082. Functional annotation revealed that 97.14% of the predicted genes matched homologous sequences in the NCBI Non-Redundant Protein Sequences Database, with 42.19% assigned to KOG and 46.67% linked to PFAM protein families. KEGG pathway analysis identified core metabolic pathways, including glycolysis (61 genes), the tricarboxylic acid (TCA) cycle (36 genes), and the pentose phosphate pathway (39 genes). Advanced annotation identified 272 virulence genes in Virulence Factors of Pathogenic Bacteria (VFDB) SetA and 527 potential virulence genes in SetB, which are involved in toxin biosynthesis and host interactions. Furthermore, 163 antibiotic resistance genes, including ATP-binding cassette (ABC) transporters and *β*-lactam resistance mechanisms, were annotated via the CARD database. The genome also encoded 700 carbohydrate-active enzymes (CAZy), primarily glycoside hydrolases (GHs) and glycosyltransferases (GTs), suggesting a robust capacity for carbohydrate metabolism.

**Figure 2:**
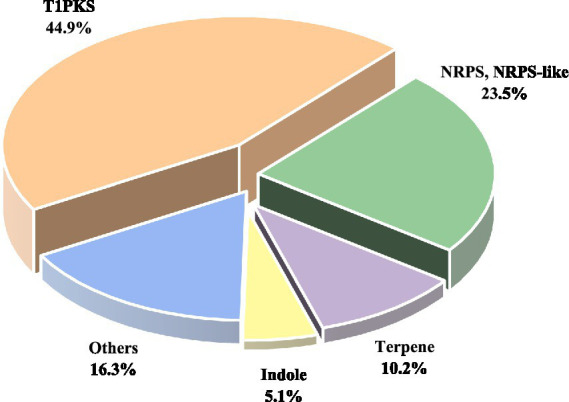
Distribution of different types of BGCs in *D. kyushuensis* ZMU-48-1.

AntiSMASH analysis revealed that *D. kyushuensis* ZMU-48-1 contains 98 BGCs (Figure 2), primarily including type I polyketide synthases (T1PKS, ~50%), non-ribosomal peptide synthetases (NRPS and NRPS-like, 23 clusters), terpenes (10 clusters), indoles, RiPPs, and hybrid BGCs (e.g., T1PKS + NRPS) (Supplementary Table S4). Several clusters showed high similarity to known pathways. Cluster 41 (T1PKS) shares 60% similarity with the altermapyrone pathway, whereas cluster 54 (Indole) shows 66% similarity to the sespendole pathway, suggesting the potential production of polyketide antibiotics and indole alkaloids, respectively. Cluster 60 (T1PKS) shows 22% similarity to the monacolin K pathway, implying cholesterol-lowering potential, whereas cluster 96 (T1PKS) shares 50% similarity with the abscisic acid pathway, suggesting a role in plant hormone regulation. Several toxin-related BGCs were also identified, including cluster 15 (37% similarity to betaenone A/B/C), cluster 16 (33% similarity to depudecin), and cluster 85 (50% similarity to PR toxin), highlighting potential toxicity. Notably, approximately 60% of the BGCs presented no significant homology to known clusters, suggesting the presence of novel biosynthetic pathways. These findings indicate that *D. kyushuensis* ZMU-48-1 exhibits extensive secondary metabolic diversity, with significant potential for discovering novel bioactive compounds for pharmaceutical and agricultural applications.

### 3.3 Optimization of culture conditions using the OSMAC strategy

**Figure 3:**
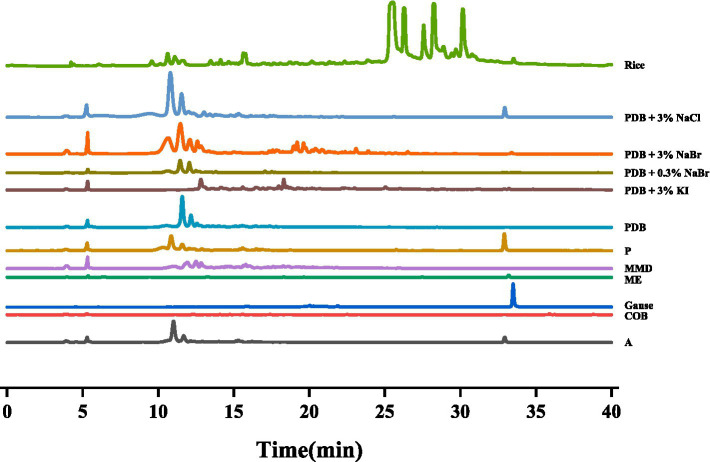
The HPLC analysis of different secondary metabolites of *D. kyushuensis* ZMU-48-1 in different medium.

**Figure 4:**
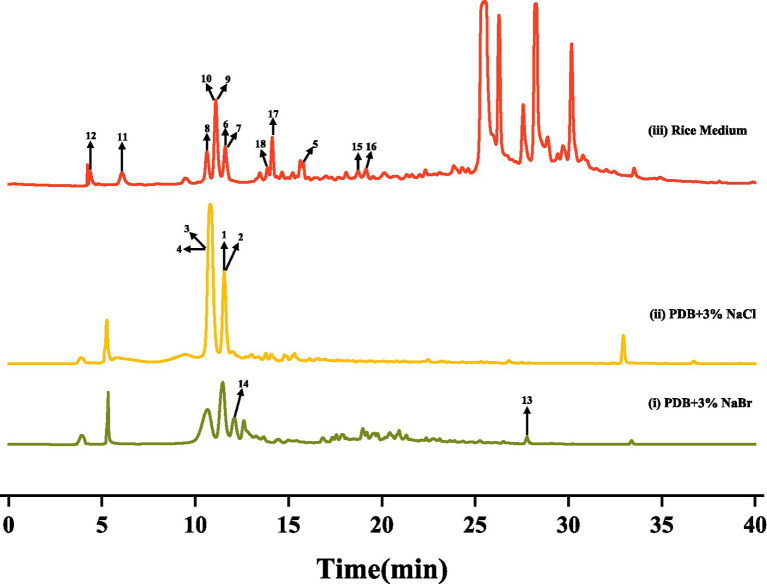
The HPLC analysis of different secondary metabolites in three medium.

**Figure 5:**
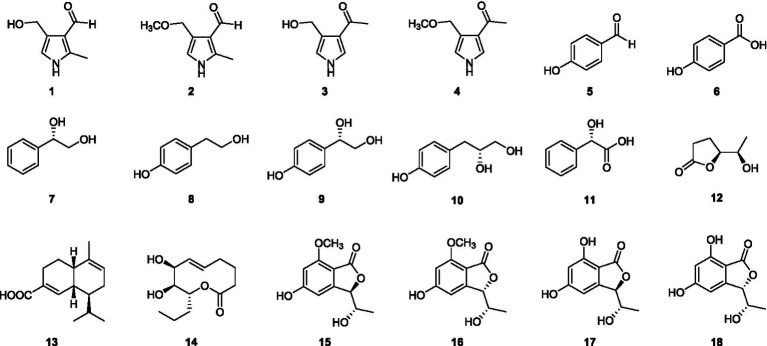
The structures of secondary metabolites (**1**–**18**) isolated from *D. kyushuensis*.

After culturing *D. kyushuensis* ZMU-48-1 in eleven liquid media and rice medium, the resulting crude metabolites were analyzed using HPLC. Based on the diversity of the metabolic peaks, PDB with 3% NaBr, PDB with 3% sea salt, and rice medium were selected as the optimal culture media for large-scale cultivation (Figure 3). Eighteen compounds were isolated from these three media (Figure 4), and their structures are shown in Figure 5.

### 3.4 Structural elucidation of the new compounds

Compound **1** was isolated as a white amorphous powder, whose HR-ESI-MS spectrum showed a pseudomolecular ion peak at *m*/*z* 140.0679 [M + H]^−^, suggesting a molecular formula of C_7_H_9_NO_2_ with four degrees of unsaturation. Its IR spectrum displayed absorption bands for hydroxyl (2,931 cm^−1^) and carbonyl groups (1743 cm^−1^). The ^1^H NMR spectrum revealed four sets of singlets (*δ*_H_ 9.79, 6.63, 4.61, 2.49) with an integral area ratio of 1:1:2:3. The ^13^C and DEPT NMR spectra revealed seven carbons, including one methyl (*δ*_C_ 11.3), an oxygenated methylene (*δ*_C_ 58.1), two methyne (an aldehyde carbon at *δ*_C_ 187.8 and an olefinic carbon at *δ*_C_ 117.4), and three olefinic quaternary carbons (*δ*_C_ 120.9, 125.9, 143.3). The above data revealed three substituent groups in the structure, namely, an aldehyde group, an oxygenated methylene, and a methyl group. On the basis of the chemical shifts of the remaining four carbons, combined with the molecular formula and unsaturation, it can be inferred that there is a three-substituted pyrrole in the molecule. The methyl group was connected to C-2, and the aldehyde group was connected to C-3 on the basis of the HMBC correlations from H-6 to C-2 and C-3 and from H-7 to C-3 and C-4 (Figure 5). The oxygenated methylene was identified as a hydroxymethyl according to its chemical shift and molecular formula, which was connected to C-4 on the basis of the HMBC correlations from H-8 to C-3, C-4 and C-5. Thus, the structure of **1** was determined to be kyushuenine A, and its NMR data are listed in Table 1.

Compound **2** was obtained as a white amorphous powder, and its molecular formula was deduced to be C_8_H_11_NO_2_ by HR-ESI-MS at *m*/*z* 154.0864 [M + H]^+^ with an unsaturation degree of four. Its IR spectrum showed the characteristic absorption of the aldehyde carbonyl group (1741 cm^−1^). A comparison of the MS and NMR spectra of **2** with those of **1** revealed that **2** had an additional methoxyl group at C-8 instead of a hydroxyl group. The ^13^C NMR spectra of **2** and **1** are very similar, except for C-8, due to the substitution effect (OH → OCH_3_). In addition, the HMBC correlations from OCH_3_-8 (*δ*_H_ 3.36, s) to C-8 further confirmed this deduction. Therefore, the structure of **2** was determined to be kyushuenine B, and the spectral data assigned by 2D NMR are listed in Table 1.

**Figure 6:**
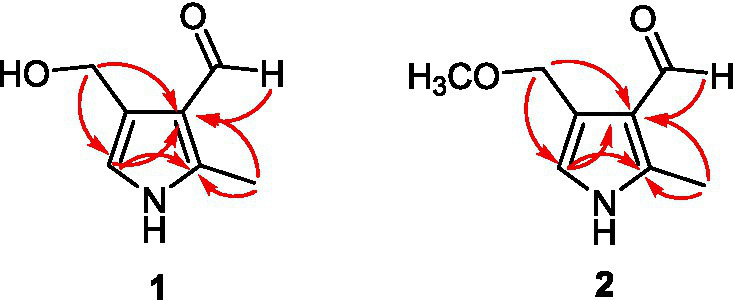
Key HMBC correlations of the new compounds **1**–**2**.

Notably, the isomers of compounds **1** and **2**, namely, verrucarin E (**3**) and 1-(4-(methoxymethyl)-1H-pyrrol-3-yl)ethan-1-one (**4**) (Zhang et al., 2022), were also isolated from this fungus (Figure 6).

On the basis of spectroscopic analyses and comparisons with the literature, the remaining known compounds were identified as eight phenolics (**5**–**11**), one lactone (**12**), one cadinene-type sesquiterpenoid (**13**), one macrolide (**14**), and four phthalides (**15**–**18**), namely, *p*-hydroxybenzaldehyde (**5**) (Wang et al., 2018), *p*-hydroxybenzoic acid (**6**) (Wang et al., 2018), (1*S*)-1′-(4-hydroxyphenyl)ethane-1′,2′-diol (**7**) (Ishikawa et al., 2003), tyrosol (**8**) (Park et al., 2011), 1*S*-(4′-hydroxyphenyl)-1,2-ethanediol (**9**) (Zhou et al., 2012), lathyroxin B (**10**) (Masi et al., 2018), (*S*)-2-hydroxy-2-phenylacetic acid (**11**) (Liu et al., 2023), 3,5-dihydroxy-*γ*-caprolactone (**12**) (Wang et al., 2022), junipertriol (**13**) (Liu S. et al., 2022), (7*S*,8*S*,9*R*)-7,8-dihydroxy-9-propyl-5-nonen-9-olide (**14**) (Rivero-Cruz et al., 2000), 5,7-dihydroxy-3-(1-hydroxyethyl)phthalide (**15**), norpestaphthalide C (**16**) (Chen et al., 2022), (3*R*,8*S*)-5,7-dihydroxy-3-(1-hydroxyethyl)phthalide (**17**), (3*S*,8*S*)-5,7-dihydroxy-3-(1-hydroxyethyl)phthalide (**18**) (Ding et al., 2008; Yang et al., 2022).

### 3.5 Antifungal activities

The antifungal activities of the compounds were assessed using the MIC method. Compound **8** exhibited an MIC of 200 μg/mL against *Bipolaris sorokiniana*, whereas the positive control carbendazim displayed an MIC of 100 μg/mL. Compound **18** demonstrated more potent antifungal activity against *Botryosphaeria dothidea*, with an MIC of 50 μg/mL, whereas carbendazim had an MIC of 12.5 μg/mL. These findings indicate that both compounds possess antifungal potential, with **18** exhibiting superior efficacies against *Botryosphaeria dothidea* (Table 2).

**Table 2:** The antifungal activity compounds **1**–**18** measured as MIC values.

Compounds	*B. sorokiniana*	*B. dothidea*	*F. graminearum*	*F. oxysporum* Schltdl	*C. gloeosporioides*	*V. mali* Miyabe & G. Yamada	*C. musae*	*A. alternata*	*C. agencrium*
	MIC, μg/mL								
**1**	>200	>200	>200	>200	>200	>200	>200	>200	>200
**2**	>200	>200	>200	>200	>200	>200	>200	>200	>200
**3**	>200	>200	>200	>200	>200	>200	>200	>200	>200
**4**	>200	>200	>200	>200	>200	>200	>200	>200	>200
**5**	>200	>200	>200	>200	>200	>200	>200	>200	>200
**6**	>200	>200	>200	>200	>200	>200	>200	>200	>200
**7**	>200	>200	>200	>200	>200	>200	>200	>200	>200
**8**	**200**	>200	>200	>200	>200	>200	>200	>200	>200
**9**	>200	>200	>200	>200	>200	>200	>200	>200	>200
**10**	>200	>200	>200	>200	>200	>200	>200	>200	>200
**11**	>200	>200	>200	>200	>200	>200	>200	>200	>200
**12**	>200	>200	>200	>200	>200	>200	>200	>200	>200
**13**	>200	>200	>200	>200	>200	>200	>200	>200	>200
**14**	>200	>200	>200	>200	>200	>200	>200	>200	>200
**15**	>200	>200	>200	>200	>200	>200	>200	>200	>200
**16**	>200	>200	>200	>200	>200	>200	>200	>200	>200
**17**	>200	>200	>200	>200	>200	>200	>200	>200	>200
**18**	>200	**50**	>200	>200	>200	>200	>200	>200	>200
**Carbendazim**	100	3.125	50	150	1.0625	12.5	6.25	125	12.5

## 4 Discussion

The integration of genome mining with the OSMAC strategy represents a paradigm shift in natural product discovery, moving beyond traditional bioactivity-guided isolation. In this study, the discovery of two novel pyrrole derivatives, kyushuenines A (**1**) and B (**2**), significantly expands the known structural repertoire of *Diaporthe*-derived metabolites. Notably, over 60% of the 98 BGCs identified through antiSMASH analysis showed no significant homology to known clusters, consistent with previous reports that highlight the predominance of cryptic and silent pathways in fungal genomes (Ziemert et al., 2016; Yee et al., 2023). These results support the hypothesis that underexplored endophytic fungi, when subjected to environmental stress or modified nutrient conditions, can activate otherwise inaccessible biosynthetic pathways.

Bioinformatic analysis indicates that biosynthetic gene BGC 8.1 (Supplementary Figure S3) shares 21% overall similarity with the known trypacidin gene cluster (Throckmorton et al., 2016; Weber et al., 2017). Further sequence comparison reveals that the PKS gene within cluster 8.1 exhibits 58.8% identity to the trypacidin PKS, suggesting that this cluster may be responsible for the biosynthesis of trypacidin analogs. Based on the structures and characteristics of the isolated compounds, we propose that BGC 8.1 is involved in the production of compounds **15**–**18**. Future investigations, including heterologous expression, are planned to validate this hypothesis. The failure to link other compounds to predicted BGCs is likely due to the incompleteness of the genome assembly and the current limitations of annotation tools. Additional molecular biology approaches will be employed to identify the BGCs responsible for the biosynthesis of the remaining metabolites.

Despite the successful activation of several cryptic BGCs through the OSMAC approach, the majority remained silent under the tested conditions. Furthermore, although certain metabolites exhibited antifungal activity, their potency was generally moderate compared to that of commercial agents such as carbendazim, suggesting the need for further structural optimization or pathway engineering. Future research should focus on transcriptomic profiling, targeted activation using promoter engineering, and heterologous expression systems to elucidate the regulatory mechanisms and functional potential of these BGCs.

Collectively, our results validate the genome mining-OSMAC combinatorial approach as a powerful and efficient strategy for accessing novel chemical entities. At the same time, they underscore the persistent challenge of converting genomic potential into metabolite production, highlighting the need for integrative methods to unlock the full biosynthetic capacity of fungal genomes.

## 5 Conclusion

This study highlights the successful application of genome mining combined with the OSMAC strategy to the endophytic fungus *Diaporthe kyushuensis* ZMU-48-1, leading to the isolation of 18 secondary metabolites, including two novel pyrrole derivatives, kyushuenines A and B. These findings expand the chemical space of *Diaporthe*-derived natural products and underscore their potential as antifungal leads.

The identification of 98 BGCs, over 60% of which show no homology to known clusters, reflects the untapped biosynthetic capacity of this strain. Environmental modulation *via* OSMAC proved to be an effective, non-genetic means of activating silent pathways.

Despite these advances, many BGCs remain unexpressed, and the bioactivity of isolated compounds is moderate. Future work should prioritize functional validation of cryptic clusters through transcriptomics, heterologous expression, and targeted activation to fully exploit the genomic potential of this promising fungal resource.

## Data Availability

The datasets presented in this study can be found in online repositories. The names of the repository/repositories and accession number(s) can be found in the article/Supplementary material.
