# Supplementary material for: Genome mining and OSMAC strategies unveil diverse secondary metabolites from the endophytic fungus Diaporthe kyushuensis ZMU-48-1 with antifungal activity
Source: Front Microbiol. 2025 Jun 10;16:1604639. doi: 10.3389/fmicb.2025.1604639 (PMC12185548; doi:10.3389/fmicb.2025.1604639)
Supplement: Supplementary file 1 [file Data_Sheet_1.docx]

Supplementary Material

Genome Mining and OSMAC Strategy Unveil Diverse Secondary Metabolites from Endophytic Fungus *Diaporthe kyushuensis* ZMU-48-1 with Antifungal Activity

**Jiaqi Zheng^+^, Haiwen Wang^+^, Xijing Wang, Siyu Zeng, Siwen Yuan ^*^, Tianpeng Yin ^*^**

***Corresponding author**: [ytp@zmu.edu.cn](mailto:ytp@zmu.edu.cn) (TPY)；[yuansiwen@zmuzh.edu.cn](mailto:yuansiwen@zmuzh.edu.cn) (SWY)

^+^ These authors have made equal contributions to the paper

**List of Supporting Information**

content

[**Figure S1: Isolation Workflow and Conditions for Compounds from *D. kyushuensis* ZMU-48-1** 3](#_Toc198765819)

[**Figure S2: Proposed Biosynthetic Pathway of Novel Pyridine Derivatives from *D. kyushuensis* ZMU-48-1** 4](#_Toc198765820)

[**Figure S3: Detailed Biosynthetic Gene BGC 8.1 of *D. kyushuensis* ZMU-48-1** 4](#_Toc198765821)

[**Table S4: ZMU-48-1 biosynthetic gene cluster statistics** 4](#_Toc198765822)

[**Figure S5: HRESIMS spectrum of compound 1** 11](#_Toc198765823)

[**Figure S6: IR spectrum of compound 1** 12](#_Toc198765824)

[**Figure S7: ^1^H-NMR (600 MHz, CD_3_OD) spectrum of compound 1** 12](#_Toc198765825)

[**Figure S8: ^13^C-NMR and DEPT (150 MHz, CD_3_OD) spectrum of compound 1** 13](#_Toc198765826)

[**Figure S9: HSQC spectrum of compound 1** 13](#_Toc198765827)

[**Figure S10: HMBC spectrum of compound 1** 14](#_Toc198765828)

[**Figure S11: ^1^H-^1^H COSY spectrum of compound 1** 15](#_Toc198765829)

[**Figure S12: HRESIMS spectrum of compound 2** 15](#_Toc198765830)

[**Figure S13: IR spectrum of compound 2** 15](#_Toc198765831)

[**Figure S14: ^1^H-NMR (600 MHz, CD_3_OD) spectrum of compound 2** 16](#_Toc198765832)

[**Figure S15: ^13^C-NMR and DEPT (150 MHz, CD_3_OD) spectrum of compound 2** 17](#_Toc198765833)

[**Figure S16: HSQC spectrum of compound 2** 17](#_Toc198765834)

[**Figure S17: HMBC spectrum of compound 2** 18](#_Toc198765835)

[**Figure S18: ^1^H-^1^H COSY spectrum of compound 2** 19](#_Toc198765836)

**Figure S1: Isolation Workflow and Conditions for Compounds from *D. kyushuensis* ZMU-48-1**

**Figure S2: Proposed Biosynthetic Pathway of Novel Pyridine Derivatives from *D. kyushuensis* ZMU-48-1**

**Figure S3: Detailed Biosynthetic Gene BGC 8.1 of *D. kyushuensis* ZMU-48-1**

**Table S4: ZMU-48-1 biosynthetic gene cluster statistics**

| **Cluster** | **Type** | **From** | **To** | **Most similar known biosynthetic gene cluster** | **Similarity** |
| --- | --- | --- | --- | --- | --- |
| **1.1** | T1PKS | 491,269 | 536,348 | NA |  |
| **1.2** | T1PKS | 935,960 | 982,905 | NA |  |
| **1.3** | Others | 2,412,955 | 2,453,872 | NA |  |
| **3.1** | NRPS-like | 326,121 | 369,778 | NA |  |
| **3.2** | T1PKS, NRPS | 1,636,595 | 1,727,085 | NA |  |
| **4.1** | T1PKS | 211,835 | 247,518 | NA |  |
| **4.2** | T1PKS | 312,776 | 355,819 | NA |  |
| **4.3** | T1PKS | 1,007,332 | 1,059,167 | NA |  |
| **5.1** | Terpene | 1,450,622 | 1,471,800 | NA |  |
| **6.1** | T1PKS | 543,453 | 588,707 | NA |  |
| **6.2** | T1PKS | 1,117,316 | 1,165,433 | NA |  |
| **7.1** | Fungal-RiPP | 201,225 | 239,515 | NA |  |
| **7.2** | Terpene | 358,423 | 379,609 | NA |  |
| **8.1** | T1PKS | 246,210 | 313,698 | Trypacidin | 21% |
| **9.1** | T1PKS | 252,781 | 302,307 | Betaenone A/B/C | 37% |
| **10.1** | T1PKS | 602,599 | 649,894 | Depudecin | 33% |
| **10.2** | Indole | 759,833 | 781,290 | NA |  |
| **11.1** | T1PKS, NRPS | 126,667 | 208,379 | Leucinostatin A/B | 10% |
| **12.1** | T3PKS | 556,749 | 598,111 | NA |  |
| **14.1** | Indole | 590,549 | 613,601 | NA |  |
| **16.1** | T1PKS | 41,790 | 86,045 | NA |  |
| **16.2** | Terpene | 103,455 | 124,510 | NA |  |
| **16.3** | NRPS, T1PKS | 253,622 | 306,608 | NA |  |
| **16.4** | T1PKS | 386,834 | 428,260 | TAN-1612/1-(2,3,5,10-tetrahydroxy-7-methoxy-4-oxo-1,2,3,4-tetrahydroanthracen-2-yl)pentane-2,4-dione desmethyl TAN-1612 | 40% |
| **16.5** | T1PKS | 451,305 | 498,713 | NA |  |
| **17.1** | T1PKS | 279,078 | 328,685 | NA |  |
| **18.1** | Terpene | 36,292 | 45,777 | NA |  |
| **18.2** | T1PKS | 226,841 | 276,224 | NA |  |
| **18.3** | T1PKS | 564,997 | 613,946 | BetaenoneA/B/C | 25% |
| **18.4** | Terpene | 673,771 | 697,619 | NA |  |
| **21.1** | T1PKS | 149,178 | 198,414 | Tricholignan A | 25% |
| **21.2** | T1PKS | 227,774 | 291,402 | Asperfuranone | 36% |
| **22.1** | T1PKS, NRPS-like | 287,621 | 360,345 | NA |  |
| **23.1** | T1PKS | 283,509 | 329,088 | NA |  |
| **23.2** | T1PKS | 529,039 | 576,761 | NA |  |
| **25.1** | T1PKS | 234,291 | 278,309 | NA |  |
| **25.2** | NRPS-like | 335,313 | 379,824 | NA |  |
| **25.3** | T1PKS | 570,766 | 618,280 | NA |  |
| **27.1** | NRPS | 180,629 | 230,534 | NA |  |
| **27.2** | NRPS-like | 277,584 | 321,401 | NA |  |
| **27.3** | T1PKS | 451,243 | 499,244 | Altermapyrone | 60% |
| **28.1** | Terpene， NRPS-like | 82,824 | 139,282 | NA |  |
| **32.1** | T1PKS | 2,252 | 29,052 | NA |  |
| **32.2** | T1PKS | 419,909 | 468,279 | NA |  |
| **33.1** | T1PKS | 99,550 | 149,999 | NA |  |
| **34.1** | NRPS-like | 415,759 | 459,582 | NA |  |
| **36.1** | Terpene | 138,068 | 161,972 | NA |  |
| **36.2** | NRPS | 267,143 | 314,047 | NA |  |
| **37.1** | Fungal-RiPP | 307 ,362 | 355,483 | NA |  |
| **41.1** | NRPS-like | 179,324 | 223,175 | NA |  |
| **44.1** | NRPS-like | 173,841 | 216,654 | NA |  |
| **44.2** | Terpene | 323,442 | 344,774 | NA |  |
| **44.3** | T1PKS | 369,296 | 415,884 | NA |  |
| **44.4** | Indole | 425,145 | 446,472 | Sespendole | 66% |
| **45.1** | T1PKS | 95,379 | 143,176 | NA |  |
| **47.1** | NRPS, T1PKS | 124,321 | 177,518 | NA |  |
| **50.1** | NRPS | 48,099 | 95,777 | NA |  |
| **50.2** | T1PKS | 337,196 | 393,142 | NA |  |
| **52.1** | T1PKS | 128,745 | 177,006 | Monacolin K | 22% |
| **53.1** | NRPS-like | 274,819 | 318,697 | NA |  |
| **56.1** | T1PKS | 34,845 | 81,503 | Cercosporin | 25% |
| **56.2** | Indole | 146,253 | 167,910 | NA |  |
| **59.1** | T1PKS, Indole | 50,229 | 106,3491 | FusaridioneA | 12% |
| **59.2** | T1PKS, T1PKS, Indole | 162,514 | 214,040 | NA |  |
| **60.1** | T1PKS | 273,073 | 320,113 | Neosartorin | 21% |
| **62.1** | NRPS-like | 88,581 | 129,475 | NA |  |
| **62.2** | NRPS-like | 152,744 | 196,316 | NA |  |
| **63.1** | NRPS | 54 ,868 | 101,214 | NA |  |
| **64.1** | T1PKS | 55,281 | 101,928 | Naphthalene | 33% |
| **66.1** | NRPS, Betalactone | 106,580 | 158,783 | NA |  |
| **68.1** | T1PKS, Terpene | 112,439 | 173,341 | NA |  |
| **69.1** | T1PKS | 175,740 | 222,532 | NA |  |
| **72.1** | T1PKS, NRPS-like | 151,951 | 199,853 | NA |  |
| **74.1** | Siderophore | 72,876 | 84,824 | NA |  |
| **75.1** | NRPS | 44,305 | 91,231 | Hexadehydroastechrome/Terezine-D/Astechrome | 37% |
| **78.1** | NRPS | 115,797 | 178,725 | NA |  |
| **80.1** | NRPS | 62,068 | 120,492 | NA |  |
| **81.1** | NRPS-like, NRPS | 13,578 | 85,199 | NA |  |
| **81.2** | NRPS-like | 118,686 | 161 ,458 | NA |  |
| **85.1** | Terpene | 160,449 | 186,420 | NA |  |
| **88.1** | NRPS | 70,144 | 114,451 | NA |  |
| **90.1** | NRPS | 101,633 | 155,177 | NA |  |
| **93.1** | T1PKS | 123,333 | 163,469 | NA |  |
| **95.1** | NRPS-like, T1PKS | 1 | 60,719 | Solanapyrone D | 33% |
| **96.1** | Terpene | 16,213 | 36, ,968 | PR-toxin | 50% |
| **99.1** | T1PKS | 18,348 | 68,727 | NA |  |
| **102.1** | T1PKS | 28,823 | 74 ,504 | NA |  |
| **111.1** | T1PKS | 55,133 | 112,371 | NA |  |
| **112.1** | NRPS-like | 19,748 | 63,011 | NA |  |
| **113.1** | T1PKS | 48,305 | 95,985 | NA |  |
| **115.1** | Indole | 73,238 | 94,614 | NA |  |
| **118.1** | T1PKS | 1 | 30,588 | NA |  |
| **120.1** | NRPS | 56,397 | 96,460 | NA |  |
| **123.1** | T1PKS | 41,660 | 89,472 | NA |  |
| **130.1** | Terpene | 28,761 | 49,956 | NA |  |
| **144.1** | T1PKS | 646 | 37,444 | Abscisic acid | 50% |
| **146.1** | T1PKS | 1 | 35,145 | NA |  |
| **157.1** | NRPS-like | 1 | 21 ,804 | NA |  |

**Figure S5: HRESIMS spectrum of compound 1**

**Figure S6: IR spectrum of compound 1**


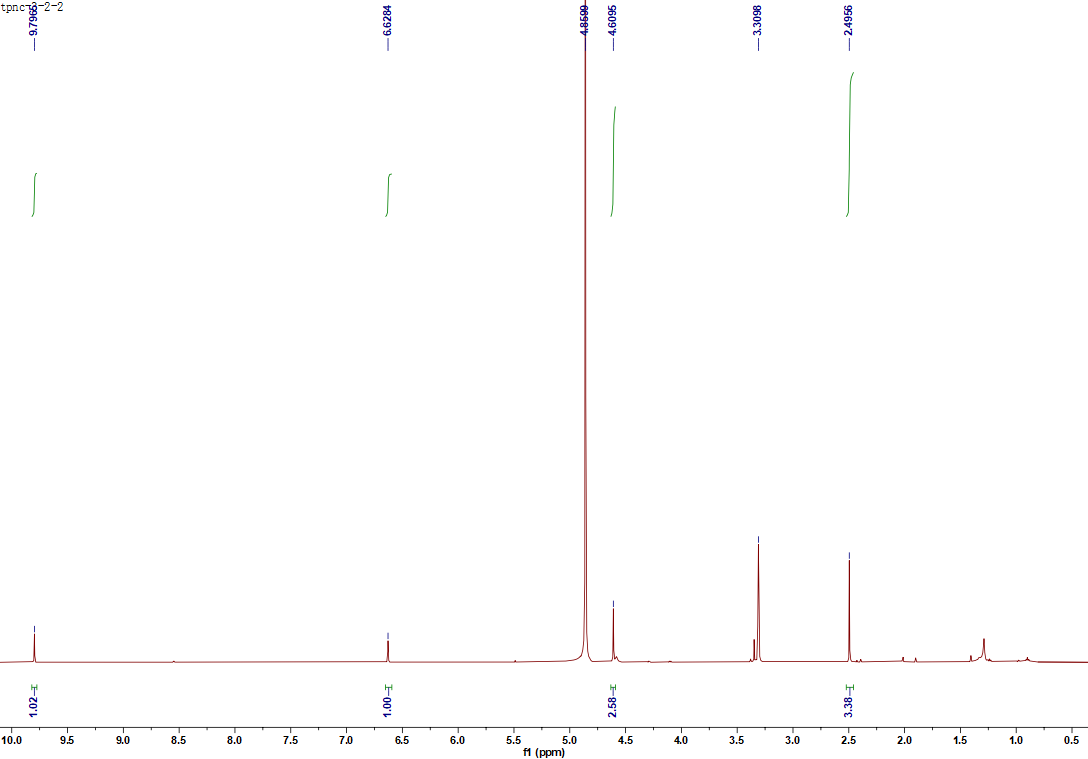


**Figure S7: ^1^H-NMR (600 MHz, CD_3_OD) spectrum of compound 1**


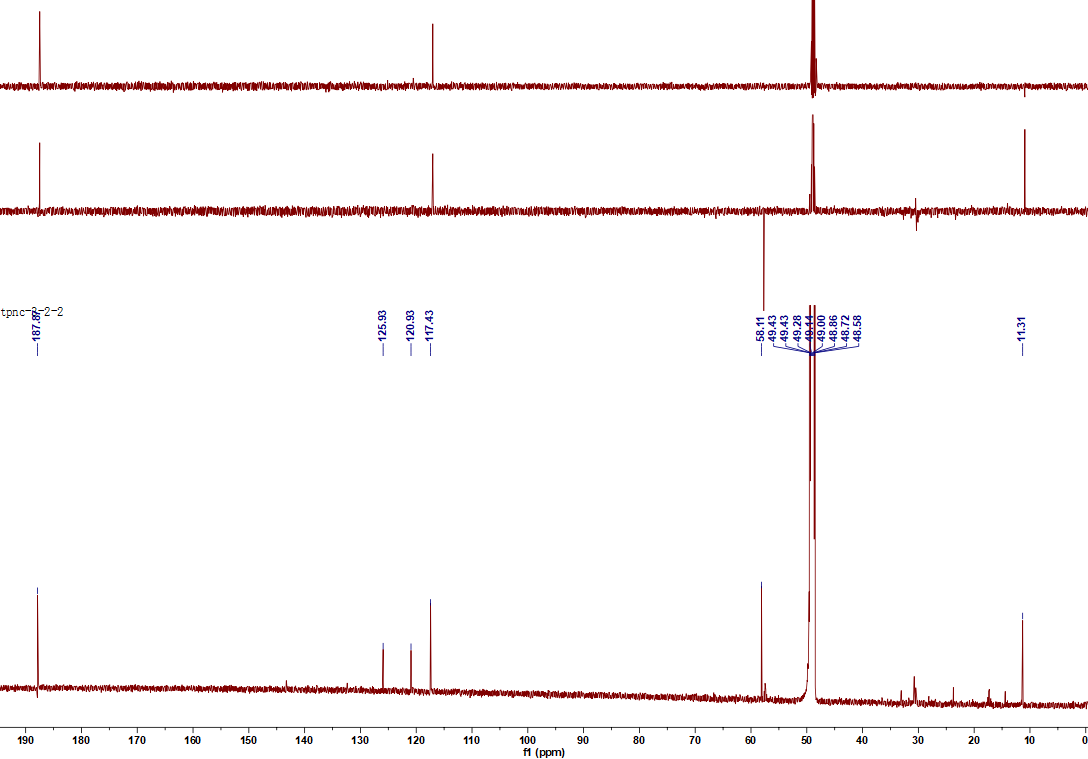


**Figure S8: ^13^C-NMR and DEPT (150 MHz, CD_3_OD) spectrum of compound 1**


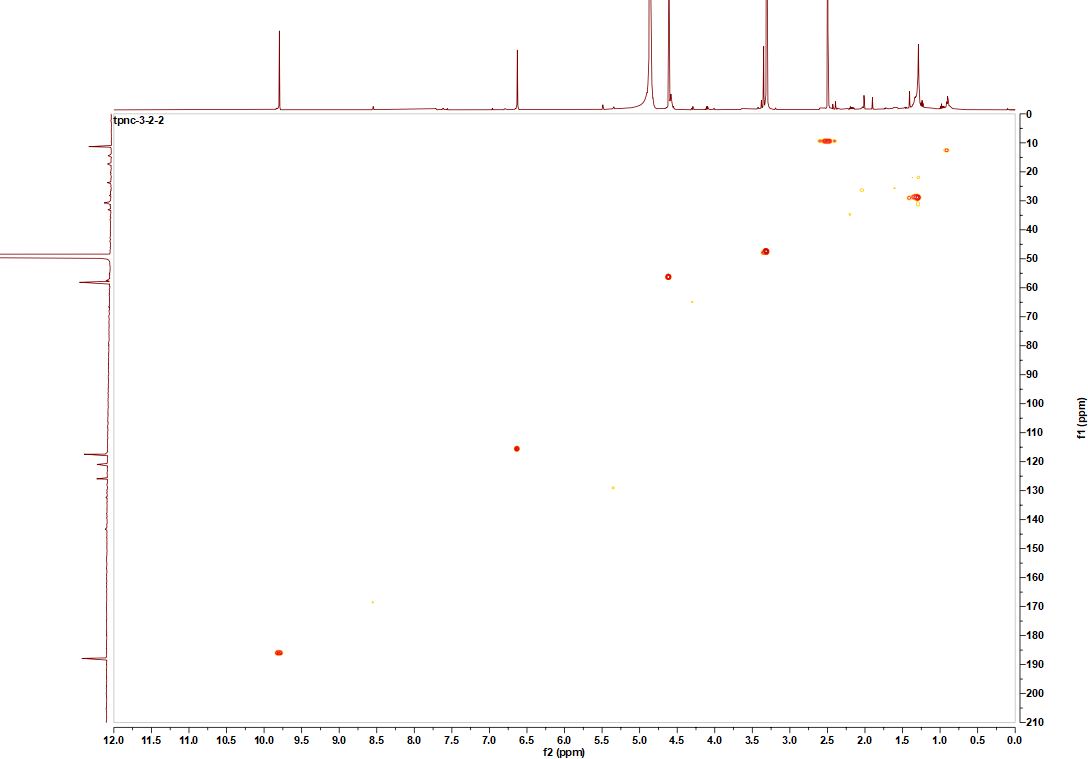


**Figure S9: HSQC spectrum of compound 1**

**Figure S10: HMBC spectrum of compound 1**

**Figure S11: ^1^H-^1^H COSY spectrum of compound 1**

**Figure S12: HRESIMS spectrum of compound 2**

**Figure S13: IR spectrum of compound 2**

**Figure S14: ^1^H-NMR (600 MHz, CD_3_OD) spectrum of compound 2**

**Figure S15: ^13^C-NMR and DEPT (150 MHz, CD_3_OD) spectrum of compound 2**

**Figure S16: HSQC spectrum of compound 2**

**Figure S17: HMBC spectrum of compound 2**

**Figure S18: ^1^H-^1^H COSY spectrum of compound 2**
